# Rapid detection of the CYP2A6*12 hybrid allele by Pyrosequencing^® ^technology

**DOI:** 10.1186/1471-2350-10-80

**Published:** 2009-08-24

**Authors:** Deborah A Koontz, Jacqueline J Huckins, Antonina Spencer, Margaret L Gallagher

**Affiliations:** 1 Division of Laboratory Sciences, National Center for Environmental Health, Centers for Disease Control and Prevention, 4770 Buford Hwy NE, Atlanta, GA 30341, USA; 2 Qiagen, Inc., 19300 Germantown Rd, Germantown, MD 20874, USA

## Abstract

**Background:**

Identification of *CYP2A6 *alleles associated with reduced enzyme activity is important in the study of inter-individual differences in drug metabolism. CYP2A6*12 is a hybrid allele that results from unequal crossover between *CYP2A6 *and *CYP2A7 *genes. The 5' regulatory region and exons 1–2 are derived from *CYP2A7*, and exons 3–9 are derived from *CYP2A6*. Conventional methods for detection of CYP2A6*12 consist of two-step PCR protocols that are laborious and unsuitable for high-throughput genotyping. We developed a rapid and accurate method to detect the CYP2A6*12 allele by Pyrosequencing technology.

**Methods:**

A single set of PCR primers was designed to specifically amplify both the CYP2A6*1 wild-type allele and the CYP2A6*12 hybrid allele. An internal Pyrosequencing primer was used to generate allele-specific sequence information, which detected homozygous wild-type, heterozygous hybrid, and homozygous hybrid alleles. We first validated the assay on 104 DNA samples that were also genotyped by conventional two-step PCR and by cycle sequencing. CYP2A6*12 allele frequencies were then determined using the Pyrosequencing assay on 181 multi-ethnic DNA samples from subjects of African American, European Caucasian, Pacific Rim, and Hispanic descent. Finally, we streamlined the Pyrosequencing assay by integrating liquid handling robotics into the workflow.

**Results:**

Pyrosequencing results demonstrated 100% concordance with conventional two-step PCR and cycle sequencing methods. Allele frequency data showed slightly higher prevalence of the CYP2A6*12 allele in European Caucasians and Hispanics.

**Conclusion:**

This Pyrosequencing assay proved to be a simple, rapid, and accurate alternative to conventional methods, which can be easily adapted to the needs of higher-throughput studies.

## Background

Cytochrome P450 (CYP) is a diverse superfamily of enzymes that catalyzes the metabolism of a wide variety of compounds, including drugs and xenobiotics [1]. *CYP2A6 *is a member of this superfamily. It was first identified as the human coumarin 7-hydroxylase enzyme [2]. *CYP2A6 *is also the primary enzyme responsible for the metabolism of nicotine to cotinine. Substantial inter-individual and inter-ethnic differences in enzyme activity are thought to be attributable to genetics as well as to environmental factors [3]. Individuals lacking a functional *CYP2A6 *gene have impaired nicotine metabolism and are therefore less prone to nicotine addiction and possibly tobacco-related cancers [4].

The *CYP2A6 *gene is located on chromosome 19, adjacent to the inactive and highly homologous *CYP2A7 *gene. Of the 17 different allelic variants in *CYP2A6 *that are reported to abolish or decrease enzymatic activity, five are the result of unequal crossover and gene conversion events between *CYP2A6 *and *CYP2A7 *[5]. The hybrid CYP2A6*12 allele is the product of such a crossover event; it contains the 5' regulatory region and exons 1–2 of the *CYP2A7 *gene and exons 3–9 of the *CYP2A6 *gene. The overall effect is a change in 10 amino acids and a reduction in enzyme activity [6].

The method currently used to genotype CYP2A6*12 involves a two-step PCR [6]. The first step (PCR I) amplifies a 2.3 kb fragment encompassing a region from 240 bp upstream of the start codon to exon 3. This method uses a forward primer that binds to a site that is identical in both *CYP2A6 *and *CYP2A7 *gene sequences. However, amplification of the *CYP2A7 *gene is prevented by use of a reverse primer that is specific for *CYP2A6 *exon 3 sequence. Thus, the primer set can amplify both CYP2A6*1 wild-type and CYP2A6*12 variant but not the *CYP2A7 *gene. The PCR I product serves as a template for two separate allele-specific PCR reactions (PCR II). Genotype determination is by gel electrophoresis of allele-specific products for CYP2A6*1 and CYP2A6*12. Although accurate and reliable, this method is not practical for use in large population screening.

The aim of this study was to design a Pyrosequencing assay to genotype the CYP2A6*12 hybrid allele that could be adapted to higher-throughput studies. The unique sequence structure of the hybrid allele lends itself to this technology. The Pyrosequencing method involves solution-based fluorescent real-time DNA sequencing whereby a sequencing primer is hybridized to a single-stranded PCR amplicon that serves as a template for sequential polymerase-catalyzed incorporation of nucleotides. Each incorporation event facilitates a cascade of enzymatic reactions that generates light displayed as a series of peaks (Pyrogram^® ^traces) representative of the nucleotide sequence. The Pyrosequencing method has been used for many applications, such as SNP genotyping [7], DNA methylation analysis [8], and gene copy number assessment [9,10].

## Methods

### Subjects

DNA samples from 90 individuals of the Polymorphism Discovery Resource (PDR) and 14 individuals of a multi-generational Centre d'Etude du Polymorphisme Humain (CEPH)/Utah family (Coriell Cell Repositories, Camden, NJ) were used to optimize and validate the Pyrosequencing assay. An additional 181 samples from the Coriell SNP500 panel and the NIGMS Human Variation panels HD24AA, HD24EC, HD24CHI, and HD07 Japanese  were genotyped using the validated Pyrosequencing assay to assess multi-ethnic variation in frequency of the CYP2A6*12 allele. The combined panels included African Americans (n = 47), European Caucasians (n = 48), Pacific Rim (n = 54), and Hispanics (n = 32). The repository retains no links to individuals, and the Coriell Institutional Review Board reviews the procedures of the repository annually. In addition, 7,159 participants aged 12 years or older from Phase 2 (1991–1994) of the Third National Health and Nutrition Examination Survey (NHANES III) [11] were screened using the Pyrosequencing assay to facilitate automation into the workflow. Use of these samples for this study was approved by the Centers for Disease Control and Prevention (CDC)/National Center of Health Statistics (NCHS) Institutional Review Board. Since the initial informed consent for use of participant blood for research did not include genetics, the CDC/NCHS Ethics Review Board approved a revised plan in 2001 which allows for the linkage of genetic results to NHANES data through the NCHS Research Data Center to ensure that confidentiality of study participant's identity is maintained [12].

### Assay Design and Validation

The Pyrosequencing assay was designed on the basis of sequences for the *CYP2A6 *and *CYP2A7 *genes obtained from the Ensembl Gene ID ENSG00000213052 and ENSG00000198077 databases, respectively [13]. The two sequences were aligned from 240 bp upstream of the start codon up to and including 25 bp of exon 3 to locate suitable PCR primer regions to specifically amplify CYP2A6*1 and CYP2A6*12 alleles but not the *CYP2A7 *gene. The forward PCR primer is located upstream of exon 2 and targets a region that is identical in both *CYP2A6 *and *CYP2A7 *sequences. The 3'-biotinylated reverse primer is specific to *CYP2A6 *sequence and binds to the beginning of exon 3. The reverse primer does not bind to the *CYP2A7 *sequence because of the presence of two neighboring nucleotide mismatches in the primer binding site. The specificities of the PCR primers and the primer melting temperatures were verified by use of the NCBI BLAST server .

**Figure 1:**
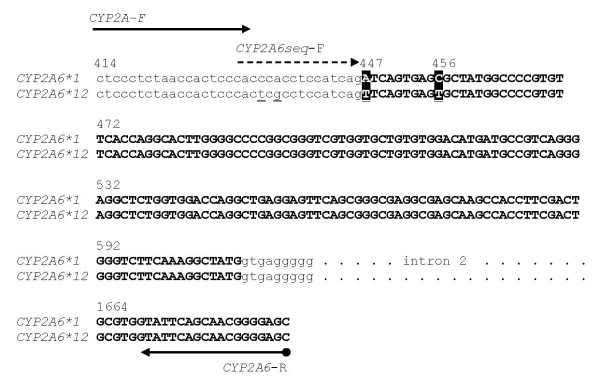
Alignment of DNA sequence for CYP2A6*1 and CYP2A6*12. Arrows indicate forward PCR and sequencing primers (5'-CTCCCTCTAACCACTCCCAC-3' and 5'-CACYCRCCTCCATCAG-3') and 3'-biotinylated reverse primer (5'-GCTCCCCGTTGCTGAATA-3'). The resulting 1274 bp PCR product is specific to CYP2A6*1 and CYP2A6*12 alleles. Exon 2 and 3 sequences are shown in bold, and the black boxes represent sequence differences between the two alleles that generate allele-specific peaks in the pyrosequencing reaction. Nucleotide numbering positions for both introns and exons are in reference to nucleotide 1, which is the A of the ATG start codon.

The internal forward sequencing primer is nested just downstream of the forward PCR primer in intron 1. It contains two degenerate nucleotides to enable the primer to hybridize to both CYP2A6*1 and CYP2A6*12-specific PCR products that differ from each other by two nucleotides at the primer binding site. The sequencing primer is positioned to interrogate two key nucleotide sites that allow for clear distinction between CYP2A6*1 and CYP2A6*12 alleles. Primer location and sequences are described in Figure 1.

PCR amplification was performed by use of the HotStarTaq Master Mix kit with Q solution (Qiagen, Valencia, CA). The 25 μl PCR reaction contained 20 ng DNA, 0.4 μM of each primer, 1× HotStarTaq Master Mix, and 1× Q-solution. Amplification was performed with initial denaturation at 95°C for 15 min, followed by 40 cycles of 95°C for 30 sec, 58°C for 30 sec, 72°C for 90 sec, and a final 72°C extension for 2 min 30 sec. PCR products (15 ul) were processed in a 96-well format for Pyrosequencing analysis by use of a Vacuum Prep Workstation, following the standard manufacturer's protocol (Biotage AB, Uppsala, Sweden). Single-stranded DNA was prepared by capture of the biotinylated strands onto steptavidin-coated Sepharose beads (GE Healthcare, Piscataway, NJ) with subsequent removal of non-biotinylated single strands. The bead-DNA complex was treated with ethanol, denaturation buffer, and wash buffer and released into a PyroMark Q96 plate containing a mixture of annealing buffer and 0.8 μM sequencing primer. The plate was incubated at 80°C on a heat block for 2 minutes, followed by a slow cool for an additional 5 minutes by turning off the heat block. The plate was transferred directly into a Biotage PSQ 96HSA System for sequence determination. Data were analyzed with PSQ HS 96 SNP software that automatically scores genotypes by using a novel SNP algorithm based on comparison of real-time sequence output (Pyrogram traces) with a theoretical histogram.

A two-phase approach was used to genotype the 90 Coriell PDR DNA samples prior to analysis by the Pyrosequencing assay. First, the conventional two-step PCR method was performed as previously described [6]. The PCR I product from this method was also directly sequenced through use of the ABI PRISM BigDye Terminator v1.1 cycle sequencing kit (Applied Biosystems, Foster City, CA). A reverse sequencing primer (5'-AGACTCTGGTCCACACTGGTCAA-3') in intron 1 was designed to capture all sequence differences between CYP2A6*1 and CYP2A6*12 in exon 1.

### CYP2A6*12 Allele Frequency Determination

The Pyrosequencing assay was used to assess multi-ethnic variation in frequency of the CYP2A6*12 allele by genotyping individuals from the African American, European Caucasian, Pacific Rim, and Hispanic population groups. For accurate assessment of allele frequency, it was important to look at *CYP2A6 *copy number, since the entire *CYP2A6 *gene can be deleted [14]. The gene copy number was determined by use of a TaqMan assay, as previously described [15]. This assay uses a FAM-labeled probe specific to *CYP2A6 *and a VIC-labeled probe specific to *CYP2A7 *that serves as an internal reference for 2 copies.

### Automation of the Pyrosequencing Workflow

The Pyrosequencing assay was used to screen 7,159 individuals from the Third National Health and Nutrition Examination Survey (NHANES III) as part of a larger study of genetic variation in xenobiotic metabolizing enzymes *(manuscript in preparation)*. To accommodate the large number of samples, robotics were integrated into the workflow. Increased genotyping throughput and accuracy of sample handling was accomplished by use of the Matrix Hydra DT (Thermo Fisher Scientific, Hudson, NH) to prepare 96-well plates of DNA. The DNA samples were allowed to dry and were stored at room temperature until needed. A Matrix WellMate (Thermo Fisher Scientific, Hudson, NH) was used for multiple dispensing steps throughout the process such as dispensing of PCR mastermix into PCR plates, dispensing of the bead mix for biotinylated PCR capture, and dispensing of the annealing buffer and sequencing primer mix into the PyroMark Q96 plate. In addition, the robotic arm of the PSQ 96HSA system was used for multiple plate testing.

## Results and discussion

**Figure 2:**
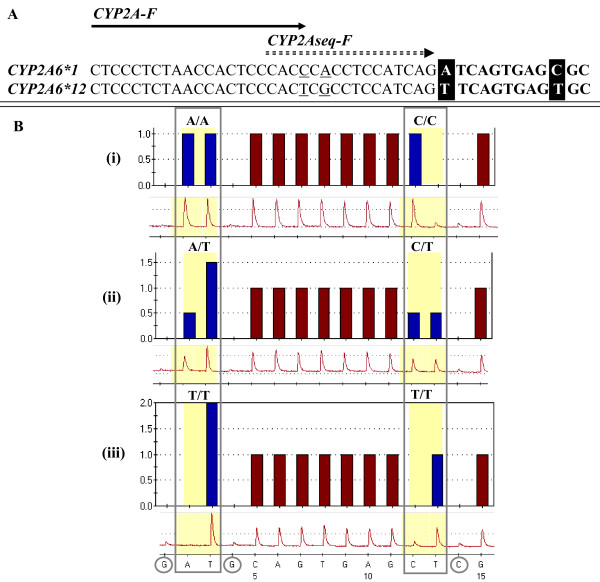
Pyrosequencing strategy and results for CYP2A6 *1 and CYP2A6*12 alleles. A) Schema of CYP2A6 *1 and CYP2A6*12 template sequences; arrows indicate forward PCR and sequencing primers, with degenerate nucleotide positions underlined. The two allele-specific nucleotides are boxed. B) Predicted histogram and pyrograms for CYP2A6*1/*1 (i), CYP2A6*1/*12 (ii), and CYP2A6*12/*12 (iii). Nucleotide dispensation order is shown at the bottom of pyrogram iii. Dispenses 2 and 3 interrogate the first allele-specific nucleotide (A in CYP2A6 *1 or T in CYP2A6*12), and dispenses 12 and 13 interrogate the second allele-specific nucleotide (C in CYP2A6 *1 or T in CYP2A6*12). Dispenses 1, 4, and 14 (circled) serve as negative background controls.

Pyrosequencing assay genotype results were 100% concordant with those obtained by conventional two-step PCR and cycle sequencing for the 90 PDR and 14 CEPH family members. The PDR is a multi-ethnic collection of unrelated individuals designed to serve as a resource for polymorphism discovery. No phenotypic or ethnic information is linked to the individual samples [16]. It also provides a robust source of DNA material for assay optimization and validation. Genotyping results generated for the CEPH multi-generational family pedigree displayed Mendelian inheritance patterns. This satisfied the validation of our Pyrosequencing assay and provided us with samples to use as positive controls for future CYP2A6*12 genotyping. Pyrosequencing strategy and representative Pyrogram results for each genotype are shown in Figure 2.

Results of the multi-ethnic variation panel are shown in Table 1. There was a higher allele frequency of CYP2A6*12 in Hispanics and Caucasians, with very low occurrence in African Americans and Asians, as has been reported previously [6,17]. Use of the TaqMan assay allowed us to calculate our CYP2A6*12 allele frequency against a known copy number background. This was of particular importance in the Pacific Rim group where 7 individuals had only one copy of the *CYP2A6 *gene. Frequencies in all ethnic groups were in Hardy-Weinberg equilibrium. None of these samples were homozygous for CYP2A6*12. However, when the assay was used to screen the 7,159 DNA samples from the NHANES III population, five Mexican-American and non-Hispanic white participants were found to be homozygous for CYP2A6*12. These results were further confirmed by the two-step PCR method.

**Table 1:** CYP2A6*12 genotype and allele frequencies by ethnicity as determined by Pyrosequencing

	Genotypes			Alleles^b^		Published CYP2A6*12 allele frequencies (# alleles tested)^ref.^
			
	*CYP2A6*	*CYP2A6*	*CYP2A6*			
Population	*1/*1^a^	*1/*12	*12/*12	CYP2A6*1	CYP2A6*12	
African American	100%	0%	0%	100%	0%	0.4% (n = 268)^17^
(n = 47)	(n = 47)	(n = 0)	(n = 0)	(n = 92)	(n = 0)	
						
European Caucasian	98%	2%	0%	99%	1%	2% (n = 1440)^17^
(n = 48)	(n = 47)	(n = 1)	(n = 0)	(n = 94)	(n = 1)	
						
Pacific Rim^c^	100%	0%	0%	100%	0%	0% (n = 194)^6^
(n = 54)	(n = 52)	(n = 0)	(n = 0)	(n = 97)	(n = 0)	0.3% (n = 354)^17^
						
Hispanic^d^	90.60%	9.40%	0%	95.30%	4.70%	2.2% (n = 184)^6^
(n = 32)	(n = 29)	(n = 3)	(n = 0)	(n = 61)	(n = 3)	

^a ^CYP2A6*1 refers to alleles without CYP2A6*12. ^b^Number of alleles based on copy number for *CYP2A6*. There were individuals with only one copy of *CYP2A6 *in African Americans (n = 2), European Caucasians (n = 1), and Pacific Rim (n = 7). Two individuals from the Pacific Rim were homozygous for the *CYP2A6 *gene deletion and were not included in the frequency data. ^c^The Pacific Rim includes Japanese, Chinese, Taiwanese, Melanesian, and Indo Pakistan. ^d^The Hispanic population is representative of the Mexican-American community of Los Angeles for this study and of a region in northern Spain for the referenced published study.

The addition of robotics into the workflow to facilitate screening of the NHANES III DNA samples greatly streamlined the process. Because of the 96-well format, the Pyrosequencing technology is amenable to a high degree of automation. Use of the robotic arm of the 96HSA system allowed for the transfer of up to ten 96-well plates for unattended operation. Data acquisition and analysis of each batch of ten plates subsequent to placement into the robotic mechanism was obtained in less than 2 hours.

In summary, we developed a single PCR-based Pyrosequencing assay for rapid detection of the CYP2A6*12 hybrid allele. Similar to conventional DNA sequencing, Pyrosequencing assays generate sequence data but without the need of a cycle sequencing step, expensive fluorescent dyes, or gel matrix; moreover, data are accessible in real-time. The assay, which is dependent upon amplification of highly specific PCR templates for wild-type and hybrid alleles, generates reliable substrates by which one can unequivocally identify CYP2A6*12 alleles.

## Conclusion

A two-step PCR is the predominant method of detection of the CYP2A6*12 hybrid allele. The Pyrosequencing assay presented here offers a rapid alternative to the significantly longer conventional method. The integration of liquid handling robotics into the workflow helps reduce errors and further streamlines the process. This approach provides a substantial decrease in laboratory effort and can help facilitate analysis of this variant in large populations used in epidemiological and pharmacogenomic studies.

## Competing interests

The authors declare that they have no competing interests.

## Authors' contributions

DK conceived of the assay design and implementation. JH and AS performed the experimental analyses. DK and MG interpreted the results and drafted the manuscript. All authors read and approved the final manuscript.

## Pre-publication history

The pre-publication history for this paper can be accessed here:


